# An Acute Bout of Soccer Heading Subtly Alters Neurovascular Coupling Metrics

**DOI:** 10.3389/fneur.2020.00738

**Published:** 2020-07-24

**Authors:** Jonathan D. Smirl, Dakota Peacock, Alexander D. Wright, Kevin J. Bouliane, Jill Dierijck, Joel S. Burma, Michael Kennefick, Colin Wallace, Paul van Donkelaar

**Affiliations:** ^1^Concussion Research Laboratory, University of British Columbia, Kelowna, BC, Canada; ^2^Faculty of Kinesiology, University of Calgary, Calgary, AB, Canada; ^3^Sport Injury Prevention Research Centre, University of Calgary, Calgary, AB, Canada; ^4^Human Performance Laboratory, University of Calgary, Calgary, AB, Canada; ^5^Hotchkiss Brain Institute, University of Calgary, Calgary, AB, Canada; ^6^Integrated Concussion Research Program, University of Calgary, Calgary, AB, Canada; ^7^Alberta Children's Hospital Research Institute, University of Calgary, Calgary, AB, Canada; ^8^Libin Cardiovascular Institute, University of Calgary, Calgary, AB, Canada; ^9^Southern Medical Program, University of British Columbia, Kelowna, BC, Canada; ^10^MD/PhD Program, University of British Columbia, Vancouver, BC, Canada; ^11^Experimental Medicine, University of British Columbia, Vancouver, BC, Canada; ^12^Faculty of Health, School of Physiotherapy, Dalhousie University, Halifax, NS, Canada

**Keywords:** repetitive football/soccer heading, sub-concussive impacts, cerebral blood flow, sport concussion assessment tool 3, SCAT3

## Abstract

**Objective:** The current investigation examined how a bout of soccer heading may impact brain function.

**Design:** Semi-randomized crossover cohort.

**Setting:** Controlled soccer heading.

**Participants:** Seven male soccer players (24.1 ± 1.5 years).

**Intervention:** 40 successful soccer headers were performed in 20 min (25 m, launch velocity ~80 km/h). X2 xPatch recorded linear and rotational head accelerations during each impact. A contact control “*sham”* condition – ball made body contact, but not by the head; and a no activity time “*control”* condition were also completed.

**Main Outcome Measures:** Posterior and middle cerebral artery (PCA and MCA, respectively), cerebral blood velocity (CBV) was recorded during a visual task (neurovascular coupling: NVC) alongside SCAT3 symptoms scores pre/post a controlled bout of soccer heading.

**Results:** Cumulative linear and rotational accelerations were 1,574 ± 97.9 g and 313,761 ± 23,966 rads/s^2^, respectively, during heading and changes in SCAT3 symptom number (pre: 2.6 ± 3.0; post: 6.7 ± 6.2, *p* = *0.13*) and severity (pre: 3.7 ± 3.6, post: 9.4 ± 7.6, *p* = *0.11*) were unchanged. In the PCA, no NVC differences were observed, including: relative CBV increase (28.0 ± 7.6%*, p* = *0.71*) and total activation (188.7 ± 68.1 cm, *p* = *0.93*). However, MCA-derived NVC metrics were blunted following heading, demonstrating decreased relative CBV increase (7.8 ± 3.1%, *p* = *0.03*) and decreased total activation (26.7 ± 45.3 cm, *p* = *0.04*).

**Conclusion:** Although an acute bout of soccer heading did not result in an increase of concussion-like symptoms, there were alterations in NVC responses within the MCA during a visual task. This suggests an acute bout of repetitive soccer heading can alter CBV regulation within the region of the brain associated with the header impacts.

## Introduction

With over 265 million players across the globe, soccer is the world's most popular sport (1). While infrequently resulting in a concussion, the subconcussive impacts associated with heading the soccer ball have been speculated to lead to potential injuries such as increases in intracranial pressure leading to retinal bleeding (2), hyphema (3), and orofacial and dental injuries (3). It has been estimated soccer players use their head to make contact with a ball ~6–16 times per game, which extrapolates to 3,500–8,500 lifetime subconcussive exposures [Reviewed in: (4, 5)] for the average amateur soccer player. While these values represent an average exposure based on the broader literature, the total number of heading events for any given player will be highly affected by player position and level of play. Furthermore, retrospective analyses have demonstrated increased rates of suspected neurodegenerative disorders among elite soccer players (6–9). Pertinently, the degree of exposure necessary to induce retinal bleeding (2), concussions (5), and any direct association to the subsequent development of chronic traumatic encephalopathy or other neurodegenerative brain diseases remains unclear (10).

Examining the role of cerebral blood flow (CBF) regulation and neurovascular coupling (NVC) responses has provided further understanding of the pathophysiological underpinnings related to both subconcussive and concussive impacts (11–13). Specifically, NVC reflects the ability to regulate CBF in response to the metabolic demand within discrete regions of the brain when they are activated (11, 14, 15). One of the most common ways to assess the NVC response is to track increases in CBF in a posterior cerebral artery (PCA) during a visual task, as these are the primary conduit vessel which supplies the visual cortex in the occipital lobe (16). Given the nature of soccer heading, there is the potential for alterations in CBF within both the frontal/temporal (supplied by middle cerebral arteries: MCA), as well as occipital (supplied by PCA) lobes of the brain (17, 18). Furthermore, the neurometabolic cascade present following a concussive impact, results in a rapid depletion of energy stores in the brain (19, 20). In the early stages of this period (hours), it is expected there will be an uncoupling of the CBF response to metabolic demands. Previous research has suggested there will be basal CBF reductions at this point (19, 20), which in turn results in larger than normal CBF increases required to match metabolic demands associated with a given cognitive task. How this response is affected by a controlled bout of subconcussive impacts is currently unknown.

The purpose of this study was to evaluate the NVC response following an acute bout of soccer heading to enhance our understanding of neurophysiological changes associated with controlled subconcussive impacts. It was hypothesized soccer heading would lead to an augmented NVC response, whereas these effects would not be observed either during contact with the soccer ball to other body regions (*sham*), nor during a non-contact *control* session.

## Materials and Methods

### Participants

This prospective cohort study enrolled 7 male soccer players (mean age 24.1 ± 1.5 years; mean body mass index 25.5 ± 1.6 kg/m^2^) with 5 + years of experience playing at the senior club or university level. Exclusion criteria included: any significant self-reported history of cardiorespiratory, cerebrovascular, neurological, severe neurodevelopmental disorders, or <5 years of experience playing soccer at an elite level. No participants were excluded on these grounds. All subjects were familiarized with testing procedures, provided written informed consent, and abstained from caffeine, exercise, and alcoholic beverages for 12 + h before testing. This study was approved by the University of British Columbia clinical research ethics board (H14-00368).

The current results are part of a larger investigation into the effects of a controlled bout of soccer heading which included assessments of blood biomarkers (21), cerebral autoregulation, cardiovascular/autonomic function, balance metrics and executive function tasks. Participants were compensated $50 CAD for each testing session, total compensation of $150 CAD across the study.

### Study Design

Participants were evaluated using a pre-test, exposure, post-test design using a NVC response protocol as described previously (16). Briefly, participants completed eight cycles of 20 sec eyes-closed, 40 sec eyes-open to a complex visual search paradigm to augment metabolic demand. Following stimulation, PCA and MCA velocity increases relative to baseline were measured using transcranial Doppler (TCD) ultrasound to characterize the NVC response.

This NVC protocol was completed prior to and ~10–15 min following a 20-min exposure to three different conditions. Testing for each condition outlined below was done on separate days in a pseudo-random order, an average of 26.1 ± 25.2 days apart:

- *Heading* – participants stood ~25 m from a JUGS machine (JUGS International, Taulatin, Oregon, USA) and performed 40 successful soccer headers in 20 min, with ~30 sec separating each trial. If a trial was unsuccessful (i.e., there was no contact by the head with the ball), another soccer ball was launched within the 30-sec window and repeated until a successful header occurred. The soccer balls employed were FIFA regulation size 5 ball, inflated to 13 psi and propelled from the JUGS machine at 77.5 ± 3.7 km/h recorded via Bushnell Velocity Speed Gun (Bushnell Outdoor Products, Richmond Hill, Ontario, Canada) in the heading group. All successful heading attempts were completed with this ball launch speed, the unsuccessful heading attempts were a result of the player not making head contact with the ball, and not a result of a reduced launch velocity. This protocol was designed to mimic one of the most consistent and controlled situations in soccer play which is likely to result in a heading situation in game play, the corner kick.
- *Sham* – This condition was identical to the heading condition except participants contacted the ball with any part of the body other than the head. This condition was performed to determine if any potential differences in the NVC response were due to making contact with the ball in general, potential “*whiplash-like”* effects (22) or if head contact was required for NVC alterations.
- *Control* – This condition is identical to the previous two, except no soccer balls were launched. Participants went to the testing area, completed 20 min of quiet rest and then returned to the laboratory. This was completed to control for any non-specific time effects on NVC responses.

### Lab-Based Instrumentation

During the laboratory assessments of NVC, participants were equipped with a three-lead electrocardiogram (ECG). Blood pressure was measured using finger photoplethysmography, with a brachial cuff to adjust finger and brachial artery height differences (Finometer; Finapres Medical Systems, Amsterdam, The Netherlands). This method has been shown to reliably assess the dynamic changes in beat-to-beat blood pressure and correlates well with intra-arterial measurements (23, 24).

2-MHz transcranial Doppler (TCD) ultrasound probes (Spencer Technologies, Seattle, WA, USA) were placed over the temporal acoustic windows to obtain cerebral blood velocity (CBV) in the vessel of interest, providing an index of CBF (25, 26). The P-1 segment of the PCA and M-1 segment of the MCA were insonated and optimized according to their signal depth, waveform, and CBV, and were confirmed with unilateral carotid compression and visual stimulation tests (25, 27). Once the cerebral arteries were confirmed, the probes were secured and locked in place with a headband (Spencer Technologies, Seattle, WA, USA). Our research group has previously demonstrated this method of indexing CBF (via CBV) to be highly reliable and reproducible in a similarly aged population, as noted via within-subject coefficient of variations of ~2–3% (28). End-tidal partial pressure of carbon dioxide (P_ET_CO_2_) was sampled by mouthpiece and monitored with an online gas analyzer (ML206; AD Instruments, Colorado Springs, CO, USA), calibrated with a known gas concentration before each collection. All data were time-aligned and collected at a sampling frequency of 1,000 Hz via an 8-channel PowerLab (AD Instruments, Colorado Springs, CO, USA) and stored for offline analysis using commercially available software (LabChart version 7.1; AD Instruments, Colorado Springs, CO, USA).

Six degree-of-freedom linear and angular head accelerations were captured at 1,000 Hz using a xPatch (X2 Biosystems; Seattle, WA) placed over the right mastoid process of participants during header and sham conditions. Acceleration events were recorded when forces exceeded the 10 *g* linear threshold. Peak linear (PLA) and peak rotational (PRA) acceleration and average impact duration were recorded for each detected event. Impacts which did not reach the 10 *g* threshold were recorded as 0 *g* for interpretations of average/cumulative impact exposure levels.

The Sport Concussion Assessment Tool – 3rd edition (SCAT3) was used to record the total number and severity of concussion symptoms before and after all exposure conditions (29). The SCAT3 includes 22 symptoms of somatic, cognitive and neurobehavioural nature with each symptom ranked on a Likert scale from 0 (absent) to 6 (severe). Total number of symptoms (range 0–22) were recorded, and total symptom severity was determined by summing the severity for each reported symptom (range 0–132). Participants were contacted in the evening following the soccer heading condition and asked to report any persistent symptoms. No players reported any symptoms at this follow up.

### Data Processing

Using the R-R intervals from the electrocardiogram for gating, beat-to-beat heart rate, mean blood pressure, and mean PCA and MCA CBV were processed in LabChart Scope View (AD Instruments, Colorado Springs, CO, USA). Data from each trial were aligned to stimulus onset (eyes open command), then averaged to generate one response per subject, per testing session. As per previous research (30), the average of the 5 sec preceding initiation (i.e., the last 5 sec of eyes closed), are reported as the baseline CBV in both the PCA and MCA. The peak velocity observed in each respective vessel obtained during the 30 sec following the eyes open command is reported in both absolute and relative terms. Area-under-the-curve to 30 sec (AUC_30_) for CBV in the PCA and MCA relative to baseline was used as an index of total activation for each paradigm.

### Statistical Analysis

Statistical analyses were conducted with SPSS v.25.0 (IBM Corp, Armonk, NY). A 3 (condition: Heading, Sham, Control) by 2 (time: pre, post) repeated measures ANOVA was conducted to examine the effect of condition and time on the neurovascular coupling measures. Where hypothesized, *a priori* simple main effects were investigated with Bonferroni corrections for condition or time. Due to the small sample size, Benjamini-Hochberg adjustments were also performed on the simple main effects to investigate the false discovery rate and potential influence of Type II errors. Data are presented as means SD. Significance was set *a priori* at *p* < *0.05*.

## Results

### xPatch Impact Sensor

During the heading condition, 98% of the successful headers were recorded over 10 *g* with an average of 40.7 ± 3.6 *g* linear acceleration and 8,097.8 ± 807.3 rad/s^2^ rotational acceleration. Combined this equates to a cumulative impact exposure of 1,574.7 ± 97.9 *g* and 313,760.6 ± 23,966.4 rad/s^2^ for linear acceleration and rotational acceleration, respectively, during the acute soccer heading exposure (Table 1). In comparison, although the sham condition did not involve any contact occurring directly to the head, 1% of these impacts registered >10 *g* on the xPatch (average linear acceleration of 3.2 ± 5.7 *g*). The average rotational acceleration during sham trials was 827.9 ± 1,424.5 rad/s^2^ rotational acceleration for cumulative impact exposures of 1,574.7 ± 97.9 *g* (linear) and 1,284.8 ± 2,453.2 rad/s^2^ (rotational: Table 1). As per study design, there were no impacts recorded >10 *g* during the control condition.

**Table 1:** Head impact data for all conditions as recorded with the xPatch sensor.

**Metric**	**Heading**	**Sham**	**Control**	***p*-value**
Total impacts (>10 g)	39.0 ± 1.5	0.4 ± 0.8	0 ± 0	<0.001
Average PLA (g)	40.7 ± 3.6	3.2 ± 5.6	0 ± 0	<0.001
Cumulative PLA (g)	1,574.7 ± 97.6	5.0 ± 9.6	0 ± 0	<0.001
Average PRA (rad/s^2^)	8,097.8 ± 807.3	827.9 ± 1,424.5	0 ± 0	<0.001
Cumulative PRA (rad/s^2^)	313,760.6 ± 23,966.4	1,284.8 ± 2,453.2	0 ± 0	<0.001
Average duration (ms)	16.4 ± 1.0	0.9 ± 1.9	0 ± 0	<0.001

*Data are presented as mean ± SD. PLA, Peak Linear Acceleration; PRA, Peak Rotational Acceleration*.

### Sport Concussion Assessment Test

**Figure 1:**
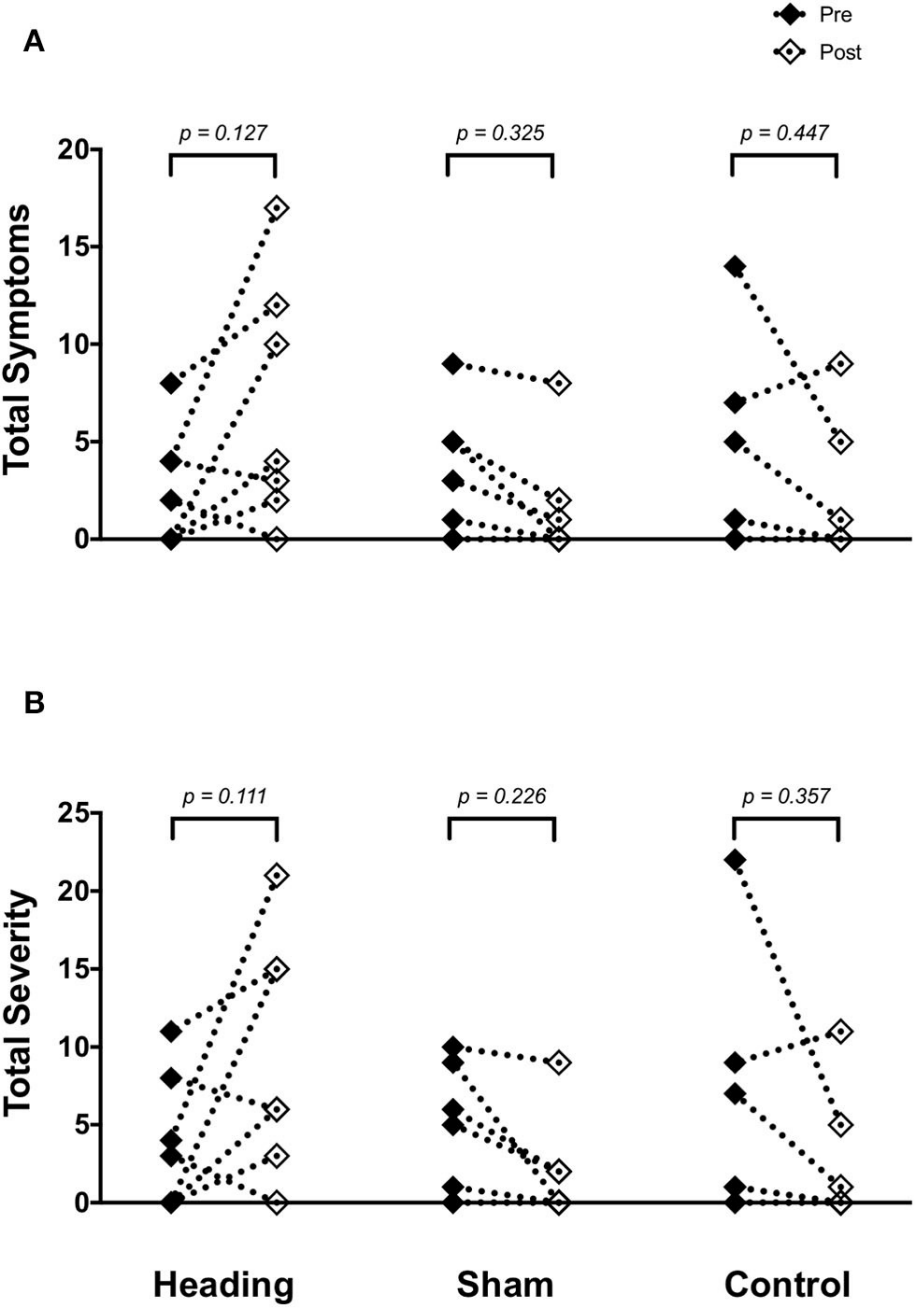
SCAT3 symptom metrics **(A)** across all three experimental conditions: heading, sham and control; and the total severity of symptoms reported on the SCAT3 **(B)** across the conditions. *p*-values represent *a priori* simple effects comparisons. Note: all individual data is reported however, some subjects reported similar values for number and severity of symptoms and are overlapped (i.e., 0 for both pre- and post-control/sham condition).

SCAT3 metrics were recorded prior to and immediately following all conditions in all participants. There were no differences in symptoms or severity of symptoms reported on the SCAT3 preceding the heading, sham or control conditions *(p* > *0.80)*. After the soccer heading, there was a greater severity of concussion-like symptoms compared with sham and control trials *(p* = *0.03)* and a trend toward an increased number of symptoms reported *(p* = *0.08)*. Overall, 71% of participants reported an increase in both the number and severity of concussion-like symptoms after soccer heading, however these alterations did not reach statistical significance (symptom number: pre 2.6 ± 3.0, post 6.7 ± 6.2, *p* = *0.13*; symptom severity: pre 3.7 ± 3.6, post 9.4 ± 7.6, *p* = *0.11*) (Figure 1). In contrast to the heading trials which showed increased symptoms, all participants in the sham heading condition and 86% of participants in the control condition reported fewer number of concussion-like symptoms and lessened severity following those conditions.

### Neurovascular Coupling Response

At pre-condition baseline, there were no differences observed in any cerebrovascular measures of interest (baseline CBV, peak CBV, total activation indexed via AUC_30_ or relative increase from eyes-closed baseline) in brain regions supplied by either the PCA (all *p* > *0.42*) or MCA (all *p* > *0.40*).

**Figure 2:**
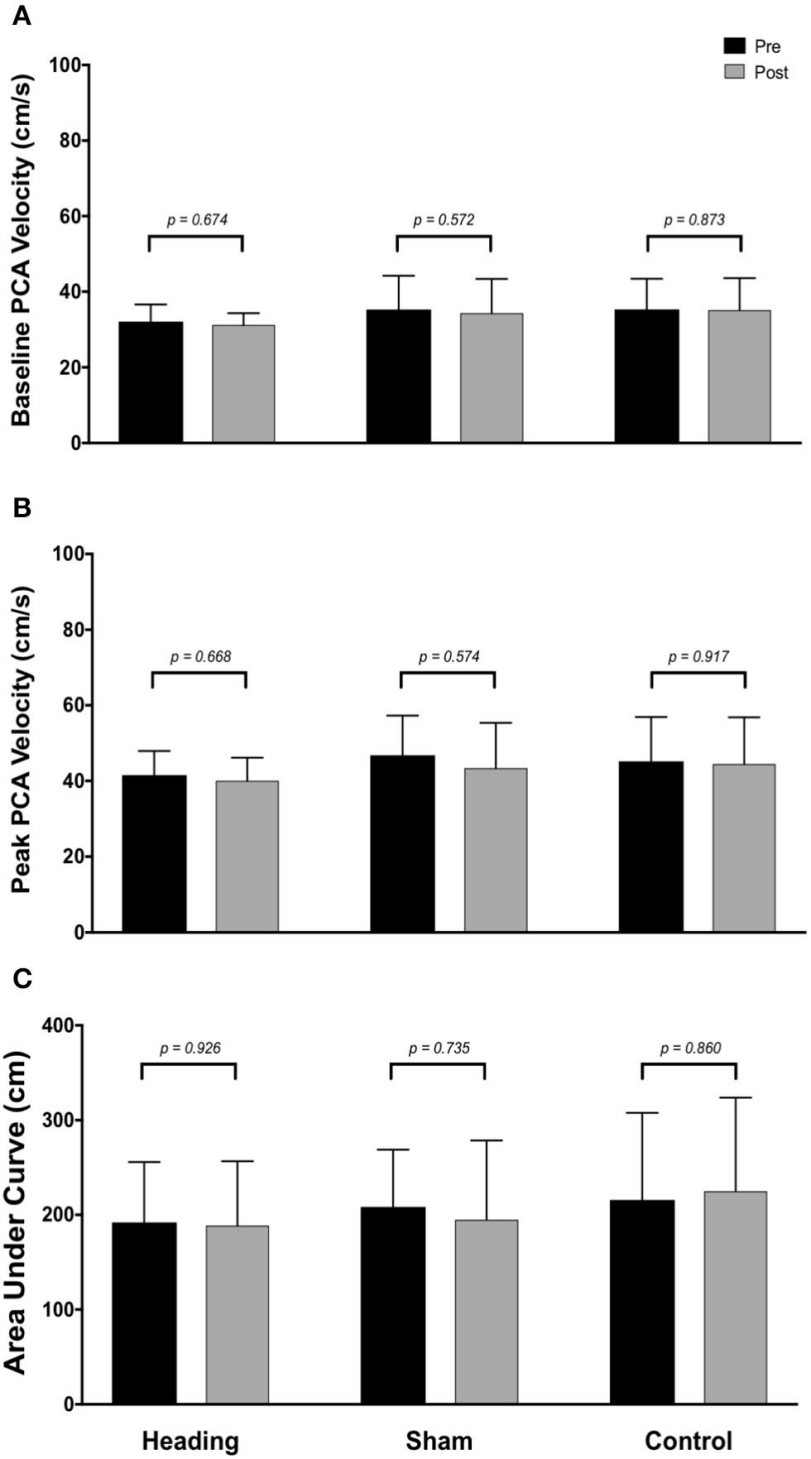
Summary of neurovascular coupling (NVC) responses to visual stimulation in the posterior cerebral artery (PCA) across participants completing the heading, sham, or control condition: **(A)** eyes-closed PCA blood velocity (baseline PCA*v*); **(B)** maximum increase in PCA*v* following visual stimulation; **(C)** area under the curve during the first 30 sec after stimulus onset. Columns represent average values of the group; error bars represent standard deviation. *p*-values represent *a priori* simple effects comparisons.

**Figure 3:**
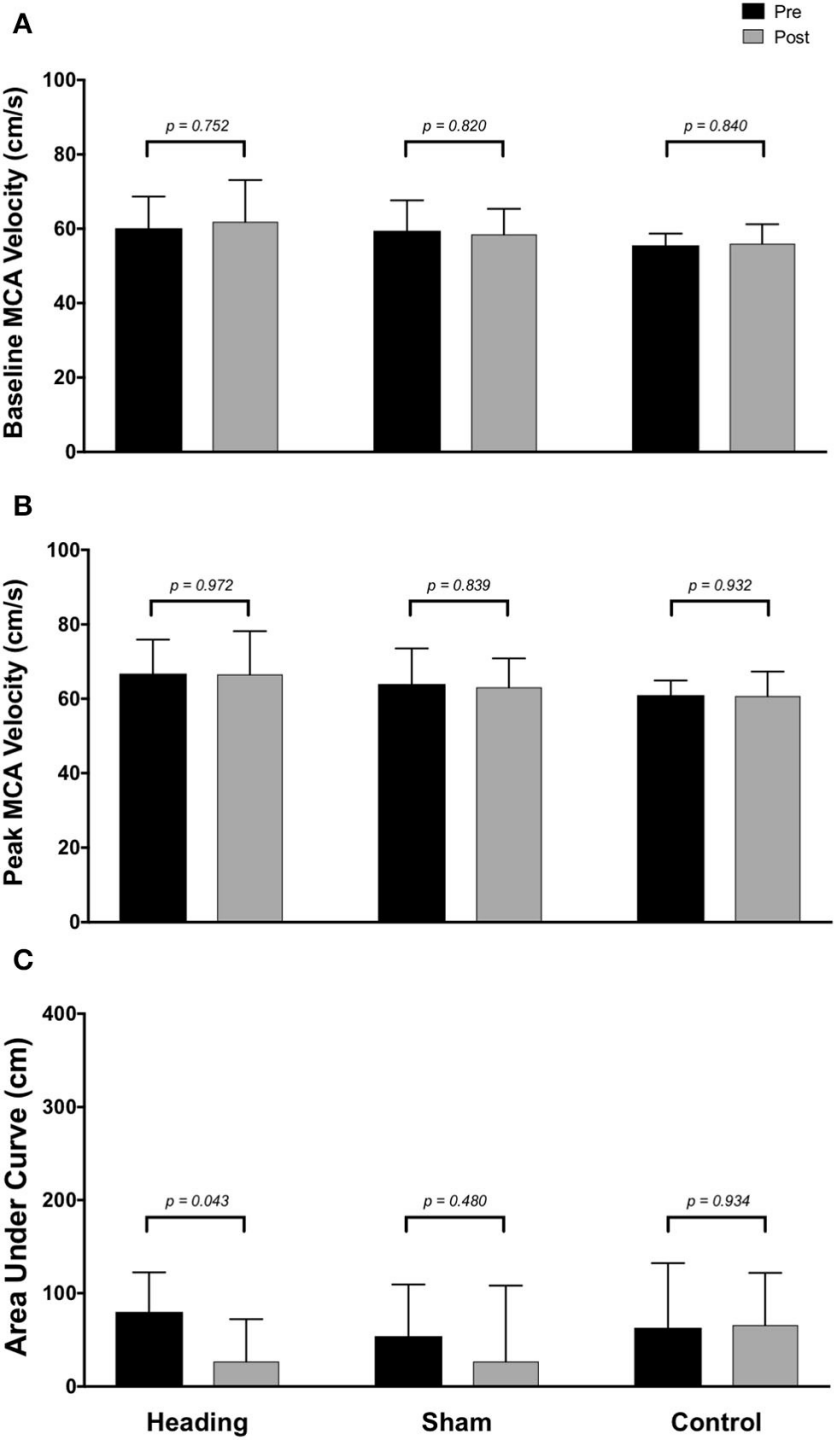
Summary of neurovascular coupling (NVC) responses to visual stimulation in the middle cerebral artery (MCA) across participants completing the heading, sham, or control condition: **(A)** eyes-closed MCA blood velocity (baseline MCA*v*); **(B)** maximum MCA*v* following visual stimulation; **(C)** area under the curve during the first 30 sec after stimulus onset. Columns represent average values of the group; error bars represent standard deviation. *p*-values represent *a priori* simple effects comparisons.

Within the posterior cerebral circulatory region, there were similarly no differences in baseline PCA CBV (*p* = *0.67*), peak PCA CBV *(p* = *0.67*), or total activation as indexed via PCA AUC_30_ (*p* = *0.93*; Figure 2). Consistent with this observation, there were also no significant changes following either the sham or control exposure detected for baseline PCA CBV (sham: *p* = *0.57*; control: *p* = *0*. *87*), peak PCA CBV (sham: *p* = *0.57*; control: *p* = *0.92*), or PCA AUC_30_ (sham: *p* = *0.74*; control: *p* = *0.86*). There were also no changes observed in the middle/anterior cerebral circulatory region following the heading, sham, or control experimental conditions for either the baseline MCA CBV (heading: *p* = *0.75*; sham: *p* = *0.82*; control: *p* = *0.84*) or peak MCA CBV (heading: *p* = *0.97*; sham: *p* = *0.84*; control: *p* = *0.93*) (Figure 3).

**Figure 4:**
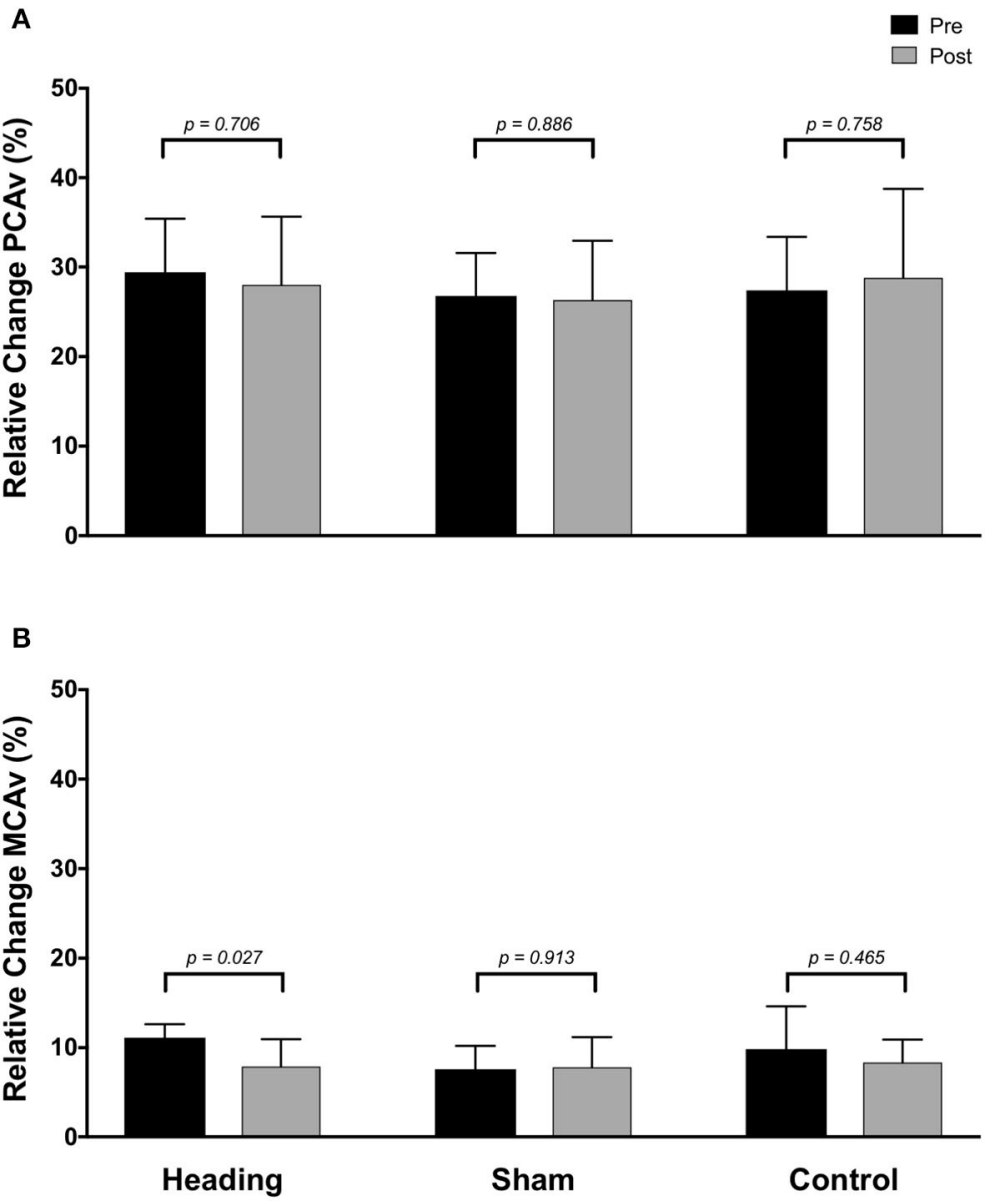
Relative increase from baseline to peak velocity of blood flow upon visual stimulation following heading, sham, or control exposures as measured in the **(A)** posterior cerebral artery and **(B)** middle cerebral artery. *p*-values represent *a priori* simple effects comparisons.

In contrast, the total activation in the regions of the brain supplied by the MCA (as indexed with MCA AUC_30_) was reduced 67% following the acute bout of controlled soccer heading (pre: 80.0 ± 42.4 cm; post: 26.7 ± 45.3 cm, *p* = *0.04*). This observation was not detected in the MCA AUC_30_ following sham (*p* = *0.48*) or control exposure (*p* > *0.93*) conditions (Figure 3). Consistent with this observation, there was a reduction in the relative increase of MCA CBV from baseline to peak velocity (11.1 ± 1.5% pre-heading; 7.9 ± 3.1% post-heading, *p* = *0.03*) following the controlled soccer heading condition, which was not observed in sham (*p* = *0.91*) or control (*p* = *0.47*; Figure 4).

There were no between group differences for MAP, HR, or P_ET_CO_2_ noted either before or after any of the experimental conditions (Table 2).

**Table 2:** Resting physiological parameters pre- and post-experimental condition exposures.

**Metric**	**Heading**		**Sham**		**Control**		***p*****-value**		
	**Pre**	**Post**	**Pre**	**Post**	**Pre**	**Post**	**Condition**	**Time**	**Condition x time**
MAP (mmHg)	95.8 ± 6.2	91.9 ± 4.4	94.2 ± 6.4	90.1 ± 3.2	91.9 ± 6.2	92.6 ± 5.3	0.604	0.155	0.439
HR (bpm)	74.9 ± 4.4	81.9 ± 6.3	75.1 ± 13.8	83.2 ± 15.6	76.6 ± 14.1	73.3 ± 11.7	0.649	0.284	0.378
P_ET_CO_2_ (mmHg)	36.8 ± 2.2	34.7 ± 2.3	36.0 ± 3.5	35.0 ± 3.0	36.8 ± 3.9	35.6 ± 4.2	t0.736	0.09	0.67

*Data are presented as mean (SD). MAP, mean arterial pressure; HR, heart rate; P_ET_CO_2_, partial pressure of end-tidal carbon dioxide*.

## Discussion

This is the first study to investigate how NVC metrics are affected by an acute bout of controlled soccer heading. The key findings from this study are 3-fold: (1) There was a trend toward an acute increase in concussion-like symptoms following an acute bout of soccer heading; (2) There were no PCA NVC response alterations; and (3) There was a reduction in total NVC activation within the MCA. Collectively, these findings suggest there are subtle NVC response alterations in the frontotemporal (supplied from the MCA) but not occipital (supplied from the PCA) regions of the brain following soccer heading.

To the authors' knowledge, the current investigation protocol included the greatest cumulative impact exposure for a controlled acute soccer heading study to date. Previous research has either used launch velocities of 40 km/h (31–39); 50 km/h (40); 65 km/h (31, 40); 80 km/h (40, 41); 90 km/h (42) and fewer soccer headers: 5 (40); 10 (31, 34, 36, 37); 12 (32, 33, 35, 41); 20 (39, 42). Despite the greater cumulative head impact exposure, the findings revealed only subtle alterations to the NVC response that were restricted to the MCA. This effect could be related to the acute nature of the sub-concussive exposure in the current study. Previous research has demonstrated differing effects on neuropsychological function based on recent vs. long-term exposures (years) to soccer heading (43, 44). However, prior research from our group has shown the PCA NVC response remains unchanged following a full season of elite contact-sport participation (13), and is disrupted following acute sport-related concussions (12). Consistent with this notion are the findings from Svaldi et al. (45, 46) who have examined cerebrovascular reactivity responses to carbon dioxide using magnetic resonance imaging measures (MRI) in female high school and collegiate soccer players. They used MRI scans at pre-season, during the first 5 weeks of the season, in the second half of the season and 1–2 months after the completion of the season while also tracking head impact exposures with the xPatch (22, 45) and demonstrated deficits in cerebrovascular reactivity in those participants with the highest cumulative accumulation of linear head impacts (those experiencing 14,487 *g* vs. 4,511 g over the season). In conjunction with the current findings, these results suggest soccer heading can dysregulate CBF control mechanisms.

Alterations observed in the MCA (Figure 3) following soccer heading were not replicated in the posterior cerebral circulation (Figure 2). One potential explanation for this finding is the relative proximity of the affected territory to the ball contact site. The MCA supplies a large anterior region of the cortex including the prefrontal cortex. Previous studies using positron emission tomography (PET) and single-photon emission computerized tomography demonstrated decreased CBF to frontal and temporal brain regions among non-athletes following mild to moderate TBI (47–49). Further, fMRI research has demonstrated decreased prefrontal cortex CBF during a working memory task among athletes after sustaining a concussion despite normal structural imaging results (50). Taken together, it is plausible an acute bout of soccer heading caused a focal change in NVC which could be due to biochemical derangement to MCA supplied brain regions. A potential mechanism of this NVC dysregulation could be related to the release of vasoactive materials from damaged neurons (i.e., CO_2_, NO, adenosine, and arachidonic acid metabolites), release of vasoactive signals from activated astrocytes, and/or direct signaling disruption within the neurovascular unit [reviewed in: (51, 52)]. These findings provide evidence that future research is warranted into additional investigations examining the effects of soccer heading on cerebrovascular regulation with respect to both acute and chronic exposure levels.

### Limitations

There are several limitations to this study. This small sample size potentially contributed to a reduced number of significant findings, particularly regarding number of concussion-related symptoms and symptom severity in the heading trial. However, given the magnitude of the head impact exposure used and the comparable sample size to previously published studies in this area (36, 40), we are confident our findings provide a meaningful contribution to this field of research. Furthermore, when the data were assessed for the false discovery rate with Benjamini-Hochberg adjustments, there were no changes to the significance reported in the results thus limiting the potential influence of Type II errors. The sham condition in the current investigation was designed to understand the effects of contact by the ball to the body, however what was not considered were the forces experienced by players when the attempt to perform a header but do not contact the ball (i.e., accelerations due to head movements, jumping, and landing). Therefore, future investigations could also include an additional sham condition which reflects this aspect of game play. Additionally, we only included male participants which limits the generalizability of our findings, as female soccer athletes sustain a greater rate of concussions per game played than male soccer athletes (53). Furthermore, females have been shown to have deficits in cerebrovascular reactivity to carbon dioxide associated with increases in head impact exposures (45, 46, 54). The research question and study design precluded the blinding of participants to exposure condition during each testing session. As such, the subjective self-reported symptoms within the SCAT3 may have led to participants being more likely to report symptoms of concussion knowing they had just been exposed to the acute bout of repeated head impacts. Potential bias introduced by the inability to blind was minimized by relying on objective outcomes (NVC response) and validated questionnaires (55). Finally, the game play situation which was mimicked in the current design (corner kick) is not entirely reflective of all potential scenarios which may result in a header, and future investigations could either employ a more diverse series of ball speeds to reflect other situations (cross, referral from the backfield) or perform a similar series of neurovascular coupling assessments across a season of play with measures performed during pre-season, mid-season and post-season with comparisons made based on player positions and number of headers they experienced as a result of game play.

A common limitation with the use of TCD is it only provides a measure of CBV within the MCA and PCA, but not CBF directly. Recently, high resolution MRI studies (56, 57) have revealed cerebral artery diameter is relatively constant when CO_2_ is within 8 mmHg of eucapnia. In the current study, end-tidal CO_2_ was maintained during all the visual stimulation protocols (Table 2), which supports the notion that CBV data presented in the current study provides a representative index of alterations in CBF. Finally, despite the excellent temporal resolution of TCD assessment of CBV, the spatial resolution is limited. Therefore, we were unable to measure changes in CBV through any of the smaller perforating branches off of the major cerebral arteries. We were therefore unable to directly quantify localized CBF, which may provide additional pathophysiological insight.

## Conclusion

This study revealed exposure to a controlled bout of subconcussive head impacts in the form of soccer heading is sufficient to change the NVC response as measured in the MCA, but not the PCA. This finding was present irrespective of a significant increase in concussion symptom number or severity following the bout of soccer heading. These findings add to our knowledge regarding the pathophysiologic mechanisms underpinning head injury in contact sports. Further investigations are warranted to investigate NVC changes within focal brain regions following a controlled bout of soccer heading to better understand the regional specificity associated with these head impacts. Furthermore, additional investigations into the cumulative effects across a soccer career of heading the ball, with respect to alterations in the NVC response are warranted.

## Data Availability Statement

The raw data supporting the conclusions of this article will be made available by the authors, without undue reservation, to any qualified researcher.

## Ethics Statement

The studies involving human participants were reviewed and approved by UBC Clinical Research Ethics Board. The patients/participants provided their written informed consent to participate in this study.

## Author Contributions

JS, PD, and AW designed the study. JS, AW, KB, JD, JB, MK, and CW collected data. JS and DP analyzed data. All authors contributed to the writing and final approval of the manuscript.

## Conflict of Interest

The authors declare that the research was conducted in the absence of any commercial or financial relationships that could be construed as a potential conflict of interest.
